# Harnessing microbiome and probiotic research in sub-Saharan Africa: recommendations from an African workshop

**DOI:** 10.1186/2049-2618-2-12

**Published:** 2014-04-16

**Authors:** Gregor Reid, Nicholas Nduti, Wilbert Sybesma, Remco Kort, Tobias R Kollmann, Rod Adam, Hamadi Boga, Eric M Brown, Alexandra Einerhand, Hani El-Nezami, Gregory B Gloor, Irene I Kavere, Johanna Lindahl, Amee Manges, Wondu Mamo, Rocio Martin, Amy McMillan, Jael Obiero, Pamela A Ochieng’, Arnold Onyango, Stephen Rulisa, Eeva Salminen, Seppo Salminen, Antony Sije, Jonathan R Swann, William van Treuren, Daniel Waweru, Steve J Kemp

**Affiliations:** 1 Lawson Health Research Institute and Departments of Microbiology & Immunology, and Surgery, University of Western Ontario, 268 Grosvenor Street, London, Ontario N6A 4V2, Canada; 2 Ministry of Agriculture, Waruhiu Agriculture training Center, P.O. Box 800, Githunguri, Kenya; 3 Yoba for Life Foundation, Hunzestraat 133-A, 1079 WB Amsterdam, The Netherlands; 4 TNO Microbiology and Systems Biology, Utrechtseweg 48, 3704 HE Zeist, and Molecular Cell Physiology, De Boelelaan 1085, 1081 HV, VU University, Amsterdam, The Netherlands; 5 Department of Pediatrics, Division of Infectious Diseases, University of British Columbia, CFRI A5-147, 950 W28th Ave, Vancouver, BC V5Z 4H4, Canada; 6 Department of Pathology, Aga Khan University Hospital, Nairobi, Kenya; 7 Taita Taveta University College, P.O. Box 635–80300, Voi, Kenya; 8 Michael Smith Laboratories and Department of Microbiology and Immunology, University of British Columbia, Vancouver, Canada; 9 Danone Nutricia Research, Uppsalalaan 12, 3584 CT Utrecht, The Netherlands; 10 School of Biological Sciences, University of Hong Kong, Pokfulam Rd, Hong Kong, Hong Kong SAR; 11 Department of Biochemistry, University of Western Ontario, London, ON, Canada; 12 Jomo Kenyatta University of Agriculture and Technology, P.O. Box 62000, (00200) Nairobi, Kenya; 13 Consultative Group on International Agricultural Research, ILRI, Nairobi, Kenya; 14 School of Population and Public Health, Faculty of Medicine, University of British Columbia, Vancouver, BC V6T 1Z3, Canada; 15 Department of Animal Production, College of Veterinary Medicine and Agriculture, Addis Ababa University, Debre Zeyte, Ethiopia; 16 Department of Reproductive Health/Biology, Institute of Primate Research, Karen, Nairobi, Kenya; 17 University Teaching Hospital of Kigali, National University of Rwanda, Kigali, Rwanda; 18 Functional Foods Forum, The Medical School, University of Turku, 20014 Turku, Finland; 19 Department of Oncology, Turku University Hospital, 20520 Turku, Finland; 20 Department of Food and Nutritional Sciences, School of Chemistry, Food and Pharmacy, University of Reading, Reading RG6 6AP, United Kingdom; 21 Department of Chemistry and Biochemistry and BioFrontiers Institute, University of Colorado at Boulder, Boulder, CO 80309, USA; 22 International Livestock Research Institute, Nairobi, Kenya

## Abstract

To augment capacity-building for microbiome and probiotic research in Africa, a workshop was held in Nairobi, Kenya, at which researchers discussed human, animal, insect, and agricultural microbiome and probiotics/prebiotics topics. Five recommendations were made to promote future basic and translational research that benefits Africans.

## Introduction

The rapid expansion of microbiome [1-5] and probiotic [6-9] research over the past 10 years and the many spin-offs providing novel insights into human and animal diseases, as well as products designed to alleviate them, have been primarily driven by sizeable funding from developed countries, especially Canada, USA and Europe.

The first study conducted in Africa was based upon 16S rRNA gene sequencing, utilizing Illumina (San Diego, CA), and revealed a microbiome of relatively high diversity in the vagina of Tanzanian women infected with HIV [10]. This was followed by an intriguing study of children from a rural village in Burkina Faso, whose high-fiber diet is similar to that eaten in early human settlements at the time of the birth of agriculture [11]; compared to European children, the latter study found a significant enrichment in Bacteroidetes and depletion in Firmicutes (*P* < 0.001), with a unique abundance of bacteria from the genus *Prevotella* and *Xylanibacter*, enriched in bacterial genes for cellulose and xylan hydrolysis. Such continental-comparative studies can be revealing in terms of understanding how the environment and diet influence health, disease and weight gain, especially since it is known that modern diet and lifestyle can cause a dramatic change to the human gut microbiome [12]. Ironically, urban regions of Africa are transitioning towards a lifestyle and fast-food diet typical of ‘Westernized’ societies, potentially bringing with it an increased risk of metabolic diseases, such as diabetes. It will be interesting to see if the gut microbiome, shown to have a higher abundance *of Bifidobacterium* and a different *Bacteroides-Prevotella* ratio at 6 months of age in resource disadvantaged Malawi compared to higher-income Finland, converts to one similar to Finland or the US [13]. Only minor differences were observed between the microbiota of subjects in Finland and Germany, emphasizing the African deviation [14]. This could have major implications as the microbiota affect energy extraction from food, possibly resulting in obesity. On the other hand, a particular nutritious diet might enhance beneficial microbiota to help lower the risk of diarrheal diseases and improve child health.

This improved knowledge of the microbial composition provides a new focus to examine the consequences of intervention studies. The importance of this is illustrated by a Swiss-led study in Ivory Coast which showed that not only was iron fortification through biscuits ineffective for iron-deficiency anemia, but it worsened the unfavorable ratio of fecal *Enterobacteriaceae* to *Bifidobacterium* and *Lactobacillus*[15]. Other microbiome and comparative microbial genome studies have contributed to our understanding of adaptation mechanism of organisms in the environment [16] and how these might have been impacted by various niches [17,18]. Of particular interest is the gut microbiome of the malaria mosquito [19], perhaps as a first step in manipulating it to lower infectivity of the parasite. Fermented foods have a long history of use in Africa, and in recent years efforts have been made to identify the microbial strains and propagate them as probiotics to confer additional health benefits [20-23]. Some studies have shown the benefits of probiotics in the gut and vagina of HIV-infected patients in Africa [24,25], while in one report a combination of probiotic and prebiotic failed to resolve acute severe malnutrition in children from Malawi [26]. Knowledge gaps remain to be investigated with respect to African subjects. For example, studies which assess the male urinary tract microbiota have been conducted in Europe [27], but in Africa studies have examined the effect of circumcision on the corona sulcus microbiota. As the interchange of male and female microbiota may have important consequences for health, coordinating such studies amongst different populations would be worthwhile.

In agreement with the sentiments brought forth by van Helden and colleagues [28] that humans, animals and the environment are inextricably linked and equal attention is needed to ensure optimal health for all, we convened a workshop in Nairobi, Kenya, at the International Livestock Research Institute (ILRA). The overall goal was to develop evidence-based concepts aimed at improving the health and wealth of people in African countries, with emphasis on mother and child, and local production and increased accessibility to nutritious and affordable products, integrated with effective education and training at community and academic levels. Three specific objectives of the workshop were: (1) discuss human, animal, insect, and agricultural microbiome as well as probiotics/prebiotics research in, or planned for, Africa; (2) engage African scientists and clinicians as well as help facilitate training focused on basic and translational research in the areas of microbiome investigations that promote sustainable, locally derived disease interventions; and (3) create new research initiatives and make recommendations to enhance African infrastructure, expertise and impact on these globally important areas.

The outcomes and conclusions of the meeting are presented here.

A cross section of researchers from across the world, with a track record in studying the microbiome and/or probiotics in Africa were invited. The meeting was limited to 40 participants for logistical reasons and to encourage discussion groups to form and come up with tangible concepts. With no funding to support travel of delegates, the reliance was upon goodwill and interest in the topic. Over 70 invitations were made to researchers based upon their publications in these areas, of which 40 accepted but eight cancelled too late for substitution, leaving a total of 32 participants from 10 countries. On day one, participants identified their interests and where they would like progress to be made. Topics for discussion were then agreed upon and day two was spent in groups to identify ways to move the topic forward.

**Figure 1:**
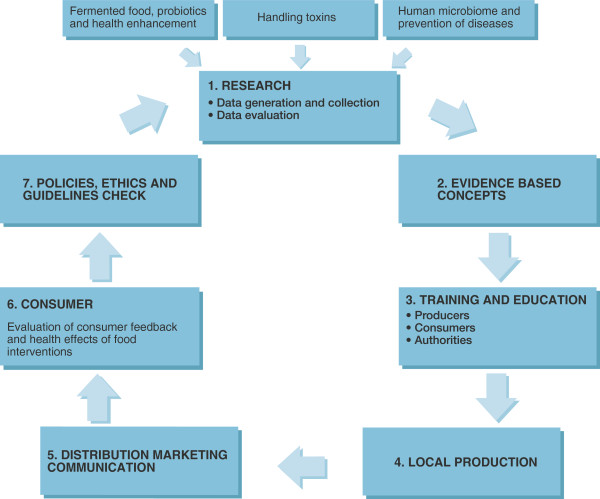
**Illustration of the model which we believe is critical for the successful expansion of microbiome and probiotic research and product development, as well as the engagement of all parties whose input is needed to the process.** Three examples of drivers for the research are given: fermented foods, the problems with inadvertent ingestion and handling of environmental toxins, and the need for remedies to treat and prevent disease.

There was collective agreement that practical translational efforts should be prioritized that embrace local involvement and focus on sustainable outcomes. Cognizant of the realities of funding and the need to develop more African expertise and infrastructure, there was a commitment from all parties to collaborate and make the proposed projects actually happen. Figure 1 provides a summary of the essential components which the participants felt were needed for success, with education, training and research being absolutely essential. The following is a summary of the ideas, projects and recommendations from these discussions. We welcome interest and contributions by members of the larger scientific community who read this article.

### The extended microbiome of African indigenous cattle

Cattle represent an integral part of society in many African countries as a source of meat and milk. Diseases of cattle have major implications for food security as well as community livelihoods and the economy. In addition, exposure of cattle to toxins, such as aflatoxins in maize or heavy metals in the soil, then become problematic for the consumer of their milk and meat, including offals. The cattle population is diversified with many breeds, but one important East African breed is the Boran which is both hardy and productive. Hypothesizing that the microbiome has an important role to play in productivity and health of cattle, five objectives were deemed important to better understand the microbiome composition.

(i) Identify the normal microbiome (bacteria, archaea and fungi) of Boran cattle, their surface parasites as well as their feed and environment in three eco-climatic zones within Kenya. This would provide insight into seasonal effects, grazing conditions and help identify microbes associated with health. All sequencing will be performed at ILRI and added to the metadata of the Earth Microbiome Project (http://www.earthmicrobiome.org/). This will also build capacity by training graduate students at various East and Central African universities.

(ii) Characterize the functional capabilities of microbial populations in the planktonic and mucosal surface of the rumen of Boran cattle. This will determine how the organisms are functioning, their by-products and impact on host immunity.

(iii) Determine the difference in microbial function when feed is contaminated with aflatoxins. The Food and Agriculture Organization estimates that 25% of the world’s food crops are affected by mycotoxins, with losses to livestock and poultry producers from aflatoxin-contaminated feeds resulting in death and effects on immune system suppression, reduced growth rates, and losses in feed efficiency. Aflatoxins are secondary metabolites produced by species of *Aspergillus*, specifically *Aspergillus flavus* and *parasiticus* fungi, which grow well on maize, peanuts and other foods under relative humidity/moisture and poor storage conditions. These compounds are highly toxic, mutagenic, teratogenic and carcinogenic, causing human hepatic and extrahepatic cancers. Regions of Kenya are known to be at high risk of aflatoxin contamination and this could adversely affect yield and increase risk for humans who consume the meat and milk. Indeed, one study of 830 animal feeds and 613 milk samples from four Kenyan urban centers showed 86% of the former were positive for aflatoxin B1 and 67% of these exceeded the Food and Agriculture Organization/World Health Organization level of 5 μg per kg maize meal [29]. Seventy two percent of the milk from dairy farmers, 84% (71/85) from large- and medium-scale farmers and 99% (88/89) of the pasteurized marketed milk were positive for aflatoxin M1. A major outbreak of aflatoxin poisoning in 2004 resulted in 317 human cases and 125 deaths as well as countless dead wildlife. The toxin can also stimulate commensal organisms to express other toxic compounds afflicting the cows [30]. The potential increase in kwashiorkor due to mycotoxin exposure is particularly relevant given the importance of the microbiome in this condition [31]. The proposed study in Boran cattle will therefore have implications for livestock productivity and human health. Of importance would be to try to quantitate aflatoxin levels, as chronic exposure is problematic over time.

(iv) Determine differences in microbial rumen composition and function in cattle associated with the presence and transmission of zoonotic pathogens. This study will identify any microbial profiles that might help the host resist infection. If beneficial profiles exist, these could form the basis for whole microbial transplantation, perhaps soon after birth. Coprophagy is commonplace in some animals, either to help develop a digestive microbiota or to act as a source of vitamins that are produced by the gut bacteria and then are part of the stool composition [32].

(v) A final component of these studies on Boran cattle would be to identify viral sequences in the transcriptome, and bank environmental DNA, RNA and rumen samples for later analysis.

The proposed studies are achievable within 2 years and would form a sound basis for many future approaches to management of livestock. These could include the relation to the effect on health and productivity by the environment, feed quality and composition, toxins, antimicrobials, heavy metals and pesticides. In addition, further insights on the reduction of methane production due to rumen bacteria could be obtained.

### The African Microbiome Project

The outstanding advances made possible through the Human Microbiome Project [33] have transformed our perception of health and disease. The current data were largely derived from North American/European subjects, and extrapolation to African subjects may be pertinent. As very few studies have been performed on microbial diversity in African humans, soil, insects, birds, fish and animals, this precludes determination of associations with health and disease. It would be presumptive to suggest skewing of microbial composition linked to negative impact on health in developed countries, are applicable in another setting. Therefore, the studies are worth replicating in Africa. Two important projects are currently underway: The American Gut Project (http://www.microbio.me/americangut/FAQ.psp) is currently surveying the diversity of 10,000 subjects (all ages, both sexes, no exclusions) in North America, and The Asian Microbiome Project is comparing diversity of subjects across 11 sites (all ages, both sexes, including a sub-focus on mothers and healthy children). It is unclear how readily concepts derived from these data sets could be extrapolated to African subjects. We hereby propose ‘The African Microbiome Project’, with the objective of determining taxonomic and functional (metagenomic) diversity of the bacteria present in the feces of 10,000 African subjects.

**Figure 2:**
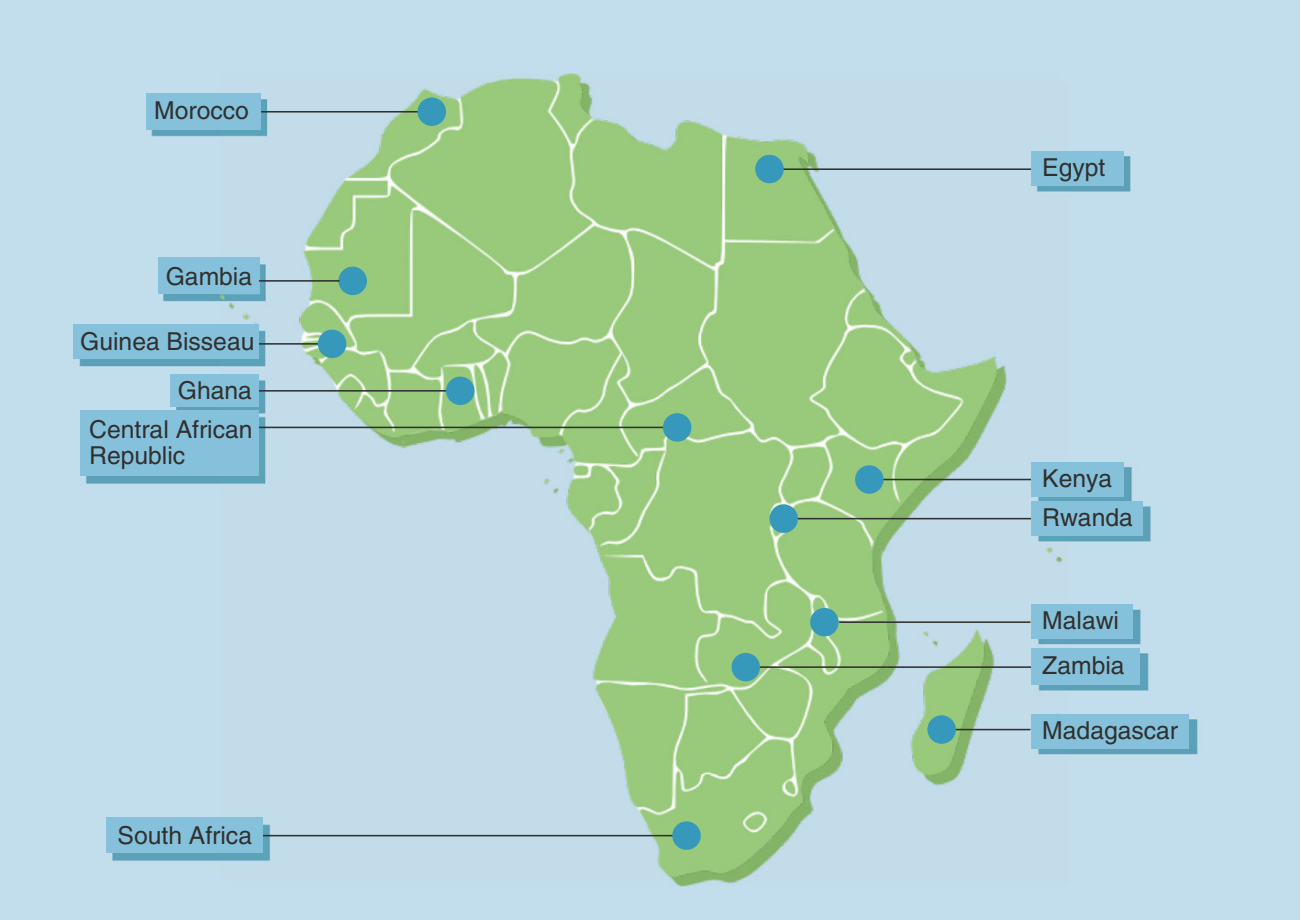
**Countries that can be targeted to perform a clinical trial on probiotics to prevent sepsis in children.** The selection is based upon existing infrastructure and collaborations, as well as extent of the sepsis problem.

To follow the design of the American and the Asian studies, the ideal would be to collect the stools across around 10 African sites (approximately 1,000 subjects per site; Figure 2). These would be processed and then shipped to ILRI in Nairobi or an equivalent site in Africa for shot-gun sequencing to determine taxonomic composition and metagenomic functional pathway representation. Extensive metadata would be collected on participating subjects using a universal form to allow association with clinically important phenotypes.

The type of metadata to be collected should include:

– For pregnant women: number of children, parity, whether term or preterm, vaginal or caesarean birth, and duration of breast-feeding.

– For infants: growth (height, weight), status at birth and need for any interventions, developmental screening and cognition, whether breast-fed or formula or other, and time of weaning.

– For all subjects: race, tribe, diet, foods, blood pressure, health history, known medications and vaccines received, HIV status, gastrointestinal problems, household size and density, exposure to animals in the home, duration at present residence, hygiene and access to toilet. Urine samples will also be acquired to allow future microbiota assessment and metabolic/functional correlative analysis [34].

The expected data resulting from such a study would not only be a tremendous resource of high-quality data on the African microbiome (taxonomic and functional), from a broad representation of multiple groups (urban/rural; different cultures; traditional and ‘Westernized’ food), but provide a rich platform for hypothesis-generating future studies using well established tools (see “Metadata and data handling” below. The next step currently under way is to identify relevant funding sources and align multi-disciplinary and multi-national teams to commence project planning in 2014.

### Metadata and data handling

The group reached consensus that samples collected in Africa should be analyzed by African trainees on site where possible. Thus, discussion centered around ways for this goal to be achieved. With regard to collecting, storing and sharing metadata, it would be useful to communicate the importance of collecting standard metadata for all samples [35]. There are a number of state-of-the-art and readily accessible resources available for Africans wishing to collect and use metadata, and the MIMARKS standard was put forward as the most widely used and general standard that should be adopted for future studies. A resource list is provided in Table 1.

**Table 1:** A list of resources

**MIMARKS provides standards on how to collect and record data**	
Tutorial	http://www.microbio.me/qiime/docs/tutorials/tutorial.html
	Examples available at: http://www.microbio.me/qiime/
	Register (free, 2-minute process) then retrieve hundreds of examples.
Tools	‘Omics’ data requires bioinformatic pipelines: QIIME, Mothur, LEfSe, MetaPhlAn, USEARCH, UPARSE, PandaSeq, and others. They are relatively complicated and non-intuitive, but the following sites are particularly helpful:
	QIIME forum: https://groups.google.com/forum/#!forum/qiime-forum
	Mothur: http://www.mothur.org/wiki/Main_Page
	SeqAnswers: http://seqanswers.com/forums/index.php
	StackExchange: http://biology.stackexchange.com/
Local clusters	As ‘Omics’ tools require computational power, it may be feasible to have a local cluster through AWS Educational Grants: http://aws.amazon.com/education/, or partner with a bioinformatics lab such as via Project Rosalind: http://rosalind.info/about/ and Project Euler: http://projecteuler.net/
Sample collection and processing protocols, bioinformatics pipelines and technical information	EMP: http://www.earthmicrobiome.org/emp-standard-protocols/
	HMP: http://www.hmpdacc.org/
	QIIME/Mothur workshops online as above, and distance workshops such as:
	STAMPS: http://hermes.mbl.edu/education/courses/special_topics/stamps.html
	QIIME tutorials: https://www.qiime.org/tutorials
	Software Carpentry: http://software-carpentry.org/
	NGS overviews: http://informaticstraining.hms.harvard.edu/
Open courses	Open courses are also available: Coursera: https://www.coursera.org/, such as Biostatistics boot camp, Machine learning, Bioinformatics methods with an MIT site being helpful. http://ocw.mit.edu/index.htm

### Clinical trial on sepsis

In sub-Saharan Africa, sepsis is the cause of at least 10% of all maternal deaths and 26% of neonatal deaths [36]. Many factors contribute to these unacceptable high rates of infection: poor or delayed diagnosis, access to care, and inadequate therapy. A recent study, so far presented orally but unpublished except in abstract form [37], of over 4,000 infants in a resource-disadvantaged setting in India reported that daily administration for 1 week of probiotic *Lactobacillus plantarum* with 150 mg fructo-oligosaccharide prebiotic in maltodextrin within 72 hours of birth reduced death or sepsis by 44%, and occurrence of pneumonia requiring antibiotics by 36%. The study appeared to be based upon an earlier encouraging controlled trial [38].

Whilst the full details of the study design and actual results still have to undergo adequate peer-review, with questions over the availability of a patent-protected formula, the workshop participants considered that a repetition of this study could be very relevant for the situation in Africa. The probiotic formulation will have to be carefully considered, taking into account the local prevailing strains as well as available resources. Besides *Lactobacillus plantarum,* another option would be *Lactobacillus rhamnosus* Yoba, basically an isolate of the GG strain, but named differently as LGG is off-patent [39], which is available in African in dried sachets.

The GG strain has been used safely in pregnant women and infants and has been shown to prevent diarrhea [40-42], indicating appropriateness for use in clinical settings. Additional design issues discussed were focused to assess not simply clinical outcome but also to begin assessing the mechanism(s) of action (presumed to be reduction of neonatal sepsis). To this end we would want to enroll the pregnant mother and sample her microbiome close to birth in a clinical trial and sample stool, saliva, skin, vagina as close as possible after birth. This would allow us to ask*:* is maternal microbiome-transfer to infant affected by probiotic intervention? We would also want to compare microbiome of the enrolled infants over the first year (stool, saliva, skin), allowing us to ask “does probiotic intervention change infant microbiome?”, and collect blood to test the impact of the probiotic intervention on innate and adaptive immune development, allowing us to ask “does probiotic intervention alter immune development, and does this correlate with increased protection?”. We would also want to collect blood, urine and milk to measure metabolome and aflatoxins of pregnant and lactating women, allowing us to investigate if the probiotic intervention alters metabolome and toxin concentration, and the amount transferred to the infant.

### Embracing African fermented foods and plant-based micronutrients

Despite a long history of a large variety of fermented foods across Africa [43,44], these are not necessarily being passed down to the next generation or incorporated in the food-based dietary guidelines. The perception conveyed by young urban people is that their generation does not have time to wait for traditional fermented foods that take a while to prepare or cost too much, or are considered inferior because of their taste and quality (as a result of non-standardized artisanal practices). The Westernization/urbanization of African society, at least in cities, was seen as a barrier to embracing most fermented foods unless they could be made available as an affordable, easy and tasty option. A means to do this was discussed, particularly around three popular Kenyan foods: Uji, Black nightshade and Murzik. Given the reported health benefits of fermented foods [45-47], the view was that consumption of these foods should be encouraged, especially since they are part of the traditional healthy diet.

The following facets, based upon bottom-of-the-pyramid principles [48], believed to be important for a new approach to fermented foods were:

• Ability to create Uji or another local food as a freshly made, fast, tasty, affordable option.

• Standardized starter cultures for consistent product quality.

• Continuous product development and product innovation based on locally available crops.

• A single message that fermented foods are beneficial to health.

• Aggressive and effective marketing, as per current fast food companies, and including use of social media.

• Nutritious and safe original local products.

• A formulation that has the potential to improve maternal-child and youth health.

• Education and training of consumers and producers based on good scientific evidence.

**Figure 3:**
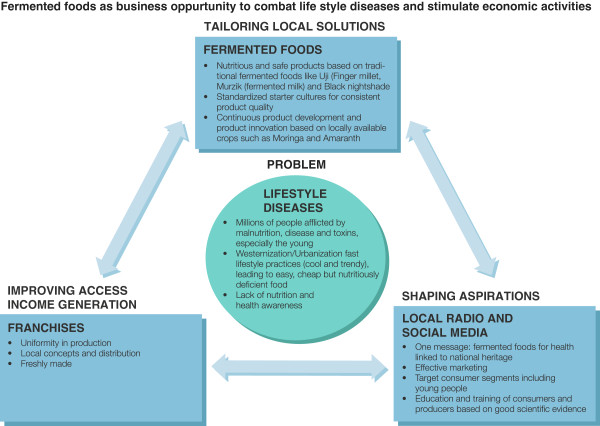
**Illustration of the problems emanating from lifestyle changes in African countries, in particular leading to changes in diet.** We believe that fermented food consumption, once a mainstay in many communities, could be re-introduced to counter poor fast foods, but to do this large efforts are needed in communicating the benefits, and social business models created that permit innovation and independence, and provide a collective message to the consumer.

In order to succeed, youth and new mothers would be targeted as a means of consumer engagement, and also as they are at high risk of obesity and diabetes, and they tend to eat more Westernized fast foods (http://apps.psychiatry.ufl.edu/Newsletters/Archive/Brownell-Kelly/FastFoodFACTS_Report_Summary.pdf). While many business models could work, the idea of franchising had appeal, where each city site could develop a novel product, share these amongst themselves and deliver a consistent single message that fermented foods are beneficial. The model is shown in Figure 3. In creating the products, microbial strain identification, storage, handling and reproducible processing would be a priority. For sustainability, cooperation amongst the franchises would allow for continuous product innovation, especially when linked with universities (different faculties), the Ministries of Agriculture and Health, and farmers. In addition, collaborations would be needed to ensure a continuous supply of raw materials and account for seasonal variations and crop growth cycles.

The addition of micronutrient rich plants was recommended, and Moringa, already used in community yogurt kitchen projects in Tanzania [49] and widely available across Africa, was considered a source of micronutrients, along with amaranth [50] and managu (*Solanum pseudocapsicum*) [51]. Inclusion of such locally sourced products will enhance collaborations between food scientists and microbiologists to create nutritious, edible and acceptable products. Similarly, local foods have been used for food supplements in nutrition intervention studies in Malawi [52,53]. A dairy product has also been developed on the basis of a traditional dish called mutandabota (made from baobab fruit pulp and sugar), and is widely consumed in Southern Africa [23].

### The challenges and opportunities related to education and training of consumers, patients, students and professionals

In order to improve the general health situation, it is key to focus on training and education of consumers, students and professionals, and national authorities. For instance, consumer education is often lacking in African countries, with many people not reaching secondary school level, and many suffering from poor cognitive function due to malnutrition. Often, information is passed from generation to generation, in some cases leading to detrimental outcomes such as early termination of breast feeding because the milk lacks ‘water’ and should be substituted by water (which is often contaminated by pathogens and causes diarrhea), sugar and other foods. The use of nutritious probiotic and fermented foods should be promoted through education of people at risk for malnutrition. This could involve training courses and/or lectures in existing school and college curricula, and educating parents and grandparents through community meetings. Potentially, university students could play a role if material was provided to aid them, and measurement tools created so they can gauge resultant change. The approach of the Early Years Collaborative (http://www.scotland.gov.uk/Topics/People/Young-People/Early-Years-and-Family/early-years-collaborative/resources) in Scotland is an example where such locally-based initiatives are tried, tested and transferred across regions as a means to alter failing processes. It is not yet focused on nutrition, but given the traditionally poor Scottish diet this will need to be addressed. Another example is the ENOUGH program (which stands for essential nutrients for optimal underpinning of growth and health) which uses multi-system approaches (physiological using omics technologies, environmental, genetic, social conditions) to reach two billion undernourished people [54]. Specifically in developing countries, the use of mass media, including social media, has been advocated to improve child-feeding practices [55].

While scarce funding for research is a problem for the entire global research community, the situation in many low-income countries is dire. At best, a small fraction of the gross domestic product is channeled to support research, which results in heavy reliance on donors and foreign agencies whose agendas do not necessarily reflect the country’s needs, but who end-up dictating national science agendas. African scientists educated abroad often return to empty laboratories and large teaching loads. In our view, the potential is enormous for many bright African students to learn these methodologies and utilize them at home to study areas of relevance to their country and population. Thus, increased national funding of research based on established priorities is clearly needed.

In Kenya, the National Commission for Science, Technology and Innovation has managed to persuade the Kenyan Government to allocate 2% of the gross domestic product to research funding which is a positive development, although it remains to be implemented. It may be a good time for researchers of high standing to support organizations like the National Commission or equivalent in other countries to provide more support of research. For example, in Canada, an advocacy organization, Research Canada, was formed to engage in influencing the public policy process directly and indirectly (http://www.rc-rc.ca/about-us). Researchers provide forums to update Members of Parliament on new advances in medical science. African scientists also could educate the public on issues of research progress in various diseases and conditions like malnutrition, and other factors that affect health and longevity.

It is no secret that scientists and policy makers have different goals, attitudes toward information, concept of time and reporting structures [56]. The lack of understanding and respect and differing views on priorities emphasizes the need to work together, have open dialogue and information sharing, and develop incentives to advance evidence-based policy and practice. Given the importance of the human and animal microbiomes and their manipulation via probiotics, efforts to convey information can only have a positive effect on the public understanding these concepts.

Another constructive suggestion is to include in a nation’s Food Guide a statement that fermented foods should be taken regularly due to their many health benefits. The rationale is supported by vast literature on the health benefits of these foods through human history.

### Increasing the impact of probiotics through food

In recent years, a practical approach has been undertaken to transfer probiotic research and products to vulnerable adults and children in Tanzania, Kenya, Rwanda and Uganda, including those who are HIV positive. The concept utilizes locally sourced food and empowers community members at the bottom-of-the-pyramid to produce probiotic yogurt. This endeavor was motivated by the eight United Nations Millennium Development Goals, established in 2000 as a moral path towards equality and health for all humankind (http://www.un.org/millenniumgoals/bkgd.shtml; Table 2). Making probiotic foods available to the poorest will contribute to all but goal 2 (achieve universal primary education) of the Millennium Development Goals [22,57-59]. An essential and sustainable target is to empower local people to establish social businesses, defined as ‘a non-loss, non-dividend company designed to address a social objective within the highly regulated marketplace of today’ [60], as well as to make probiotics (both local and international) more accessible to a larger population. The empowerment of women to create and run these social businesses has so far proved successful [38,61].

**Table 2:** The United Nations Millennium Development Goals established by a target date of 2015

**Goal number**	**Millennium goal**
**Goal 1**	Eradicate extreme hunger and poverty
**Goal 2**	Achieve universal primary education
**Goal 3**	Promote gender equality and empower women
**Goal 4**	Reduce child mortality
**Goal 5**	Improve maternal health
**Goal 6**	Combat HIV/AIDS, malaria and other diseases
**Goal 7**	Ensure environmental sustainability
**Goal 8**	Develop a global partnership for development

However, the kitchen yogurt model requires support at start-up by local medical research authorities, farmers and foreign partners, and may be unsuitable to reach the millions of people facing poverty, malnutrition, maternal and infant morbidity and mortality and infectious diseases. The delegates discussed the challenges in overcoming this barrier.

**Figure 4:**
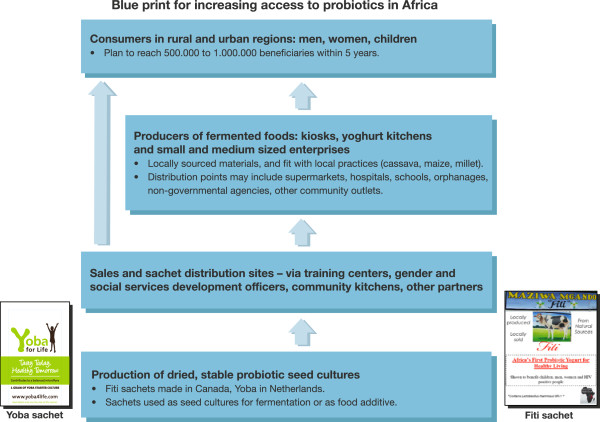
**In order to provide nutritious, safe, local foods supplemented with probiotics and affordable to the bottom-of-the-pyramid, the following model is proposed.** Existing products, Fiti yogurt in Tanzania and Kenya, and Yoba-for-life in Uganda provide stepping stones, and their distribution through community kitchens and mini-plants, and via dried sachets, with the aid of partnerships, is estimated to reach close to one million people within 5 years.

One idea is to provide as many resource disadvantaged people as possible with access to probiotic fermented foods via affordable sachets containing shelf-stable beneficial bacteria and micronutrients. The sachet contents can be inoculated into milk to make yoghurt or added to other foods and fermented, or simply sprinkled over regular food. Figure 4 shows several production and distribution channels for probiotic fermented foods using existing infrastructures, or via home-use. Collaboration across Kenya, Tanzania and Uganda could provide an impetus, with efforts conjointly being made to expand to Rwanda, Ethiopia and other neighboring countries (Table 3). The focus for the 5,000 beneficiaries of the current kitchens (10 in Tanzania, 3 in Kenya, 1 in Uganda) is mothers, children, and HIV/AIDS patients. To attain a goal of 500,000 recipients within 5 years, the products will have to reach the broader population and take account of local dietary habits. This might be via goat and camel milk in Ethiopia, or through cereals in other regions.

**Table 3:** Examples of different approaches for three countries that collectively lead to increased availability of probiotic food, including for the very poorest in society; the key engagement of partners is vital

**Where**	**Who**	**What**	**How**	**For**
Kenya	• Teachnical University of Kenya, Jomo Kenyatta University of Agriculture and Technology	• Food Science and Technology, and new product development	• Local yoghurt kitchens	• People with HIV/AIDS
	• Ministry of Agriculture, and Ministry of Gender and Social Services	• Bottom of pyramid business development	• Train-the-trainer workshops	• People exposed to aflatoxins and other population
		• Local production of Fiti yogurt	• *Lactobacillus rhamnosus* GR-1 anti-infective and immunomodulatory effects	• General population
		• Distribution networks for sachets	• *Weissella spp.* NN-20 detoxication effects	
Tanzania	• Western Heads East internship program	• Local production of Fiti yogurt	• Local yoghurt kitchens	• People with HIV/AIDS
	• African Probiotic Yogurt Network	• Supplemented with Moringa and potentially other micronutrient-rich foods	• Train-the-trainer workshops	• General population
	• National Institute for Medical Research support	• Distribution networks for sachets	• Addition of Moringa	
Uganda	• Yoba for Life Foundation	• Local production of probiotic Yoba	• Local production plants	• General population
	• Uganda Industrial Research Institute	• Food Science and Technology product development	• Using existing infrastructures	
	• Makarere University	• Bottom of pyramid social business development	• Train-the-trainer workshops	
	• Heifer International	• Incubator programs	• *Lactobacillus rhamnosus* Yoba for gut health	
	• Local dairy producers	• Distribution networks for sachets		

The partnerships form critical components of the set-up. Developed countries provide training for personnel and laboratory resources to perform microbiome analysis, genome sequencing, and probiotic research. In Africa, facilities such as at ILRI provide a means for 16S sequencing analysis. Collaborations can employ dissemination of information about foods and their health benefits, book-keeping, sanitation and food hygiene. This can also allow African concepts to be pursued. For example, a novel probiotic yogurt containing *L. rhamnosus* GR-1 and NN-20 created by Kenyans and Canadians is being tested in Embu, Kenya, for its ability to reduce aflatoxin poisoning from ingestion of corn and peanuts, through aflatoxin-binding properties [62-64].

Sachets have already been developed by partners Yoba-for-life (http://www.yoba4life.com), containing *L. rhamnosus* Yoba and *S. thermophilus*. Their 1 g dried contents, manufactured in The Netherlands, are added to 1 liter pasteurized milk and incubated in existing (small-scale) dairy production plants, then inoculated into 50 liters to produce probiotic yogurt. This avoids having to produce the probiotic Yoba at a separate site, as currently is required for the *L. rhamnosus* GR-1 Fiti yogurt produced by the Kenyan and Tanzanian kitchens. The Uganda Industrial Research Institute trains the personnel to set up and run their dairy plants, and one currently provides yogurt to 1,000 people each day. The plan is to reach 150,000 people within the next 2 years through growth of dairy plants. The Yoba strain has been extensively studied and shown to enhance immunity and prevent and treat diarrhea which are issues common to the Ugandan consumers living under poverty [39]. Depending on the production and sales point, the yogurt costs approximately 1,400 UGX/L (approximately US$0.56) to produce and is sold for 2,800 UGX/L (approximately US$1.12) to the final consumer, allowing a small profit for the employees at the production and distribution site. This sales price is around 13 cents for an equivalent tub of probiotic yogurt sold in Canada for over 43 cents.

This initiative, along with making sachets containing *L. rhamnosus* GR-1 and NN-20, will not be without challenges in education, product handling, control and pricing, but the potential payoff will be significant. At a minimum, re-introducing beneficial microbes into the food chain and society will counter the negative fast food, low nutrient products that have swept the market, or staple foods like rice and maize that offer little in nutrient value. Throughout this process, the unwavering aim will be to retain the social business, bottom-of-the-pyramid model [48]. While competitors exist now and will do so later, at least in the yogurt market, their products are expensive and without probiotic added value, and they will always have a market, especially as incomes increase with developments occurring in the region. But, for as long as millions of children and adults live in poverty and an environment where infectious diseases and other ailments affect them, the need for affordable, nutritional products will exist.

### Summary

The following recommendations emerged from this productive workshop.

1. Africa is on the verge of novel and exciting research on the microbiome and probiotics. We recommend that partnerships be created or expanded that enable the science, help train personnel, and translate evidence-based solutions in a continual circle of activity which integrates microbiome and probiotic research with products that benefit societies, no matter their cultural diversity.

2. Studies are critically needed to understand the microbiome of animals, soil, plants, insects and humans in the African continent, so as to create a platform for innovative advances in how health is restored and maintained.

3. Efforts are urgently required to take probiotic concepts in affordable food formulations and facilitate access to those who need them most. This will help reduce the risk of morbidity and mortality especially for women and newborns. Both the production and distribution of traditional local and international probiotics should be investigated. Probiotics are not a substitute for vaccines, certain drugs, hygiene practices and nutritious food, but until these options reach the most needy, probiotics can help save many lives and turn the societal culture back to some of the traditional foods that benefited past generations.

4. Programs are needed in primary/secondary schools and communities to embrace the healthful consumption of traditional foods and to resist dietary myths or new fads of fast, poor nutritional foods. Education at the college and university levels is needed to introduce the new field of microbiome research, and to train Africans on the complexities of ‘omics’ fields. Likewise, knowledge must be conveyed to the politicians who are the gatekeepers of resources. For that, we suggest the support for Research Councils composed of respected and innovative African researchers.

5. Lastly, African governments should invest in research on the microbiome and probiotics with tangible funding that provides their scientists, physicians and other researchers with the facilities and operating grants to train students, and develop community-based solutions to community-based problems. By doing so, the return on investment will far outweigh the output, with savings in healthcare and development of new businesses and products for exportation. It is no longer an option to rely on foreign philanthropy, when Africans have the knowledge, are keen to acquire the training and already show the determination to lead the field not follow it. None of the Millennium Development Goals can be reached without such an investment and without the encouragement of two-way partnerships.

## Abbreviations

ILRI: International Livestock Research Institute.

## Competing interests

The authors declare that they have no competing interests.

## Authors’ contributions

GR co-organized the workshop, invited the participants, chaired the meeting, coordinated the manuscript writing and made the final revisions; SK hosted the event and provided critical input into the manuscript; NN assisted with local organization and provided critical input into the manuscript; WS and RK provided assistance with the figures, with leading discussion during the workshop and critical input into the manuscript; TRK helped provide support and funding, and critical input into the manuscript; RA, HB, EMB, AE, HEl-N, GBG, IIK, JL, AM, WM, RM, AMc, JO, PAO, AO, SR, ES, SS, AS, JS, WVT, DW - all contributed to the discussions and critical input into the manuscript. All authors read and approved the final manuscript.
